# Regular exercise is associated with emotional resilience to acute stress in healthy adults

**DOI:** 10.3389/fphys.2014.00161

**Published:** 2014-05-01

**Authors:** Emma Childs, Harriet de Wit

**Affiliations:** Human Behavioral Pharmacology Laboratory, Department of Psychiatry and Behavioral Neuroscience, The University of ChicagoChicago, IL, USA

**Keywords:** physical activity, stress, TSST, heart rate, blood pressure, cortisol, mood

## Abstract

Physical activity has long been considered beneficial to health and regular exercise is purported to relieve stress. However empirical evidence demonstrating these effects is limited. In this study, we compared psychophysiological responses to an acute psychosocial stressor between individuals who did, or did not, report regular physical exercise. Healthy men and women (*N* = 111) participated in two experimental sessions, one with the Trier Social Stress Test (TSST) and one with a non-stressful control task. We measured heart rate, blood pressure, cortisol, and self-reported mood before and at repeated times after the tasks. Individuals who reported physical exercise at least once per week exhibited lower heart rate at rest than non-exercisers, but the groups did not differ in their cardiovascular responses to the TSST. Level of habitual exercise did not influence self-reported mood before the tasks, but non-exercisers reported a greater decline in positive affect after the TSST in comparison to exercisers. These findings provide modest support for claims that regular exercise protects against the negative emotional consequences of stress, and suggest that exercise has beneficial effects in healthy individuals. These findings are limited by their correlational nature, and future prospective controlled studies on the effects of regular exercise on response to acute stress are needed.

## Introduction

The Centers for Disease Control and Prevention (CDC) and the American College of Sports Medicine (ACSM) recommend that American adults exercise for at least 30 min on most days to improve their health and quality of life (Pate et al., 1995; Haskell et al., 2007). Indeed, clinical trials have shown that regular exercise is an effective treatment for disease, including physical ailments e.g., cardiovascular disease (Elrick, 1996), and psychiatric disorders e.g., depression (Dinas et al., 2011). Further, regular exercise is frequently associated with general well-being and lower rates of mood and anxiety disorders in cross-sectional studies (Dua and Hargreaves, 1992; Slaven and Lee, 1997; Goodwin, 2003) and with improved longevity and decreased mortality in prospective studies (Kujala et al., 1998; Samitz et al., 2011). However, the mechanisms underlying the benefits of exercise are not clear. One way in which exercise may promote health is via enhanced resilience to stress, since stress exposure and chronic stress burden has been associated with physical and mental illness (McEwen, 2007).

Acute stress produces a cascade of physiological and psychological effects that are coordinated by the sympathetic nervous system (SNS) and the hypothalamic pituitary adrenal axis (HPAA, López et al., 1999; Chrousos, 2009). Soon after encountering a stressor, defined as any emotional, physical or psychological threat that perturbs homeostasis, heart rate and blood pressure increase, along with mental alertness and tension, and cortisol is released into the blood from the adrenals (Habib et al., 2001). This multidimensional stress response is extremely beneficial and serves to ready the organism to deal with the imminent threat, however when it is improperly activated it can have deleterious effects and contribute to diseases such as atherosclerosis, obesity and depression (McEwen, 2006). Intense physical activity can also be considered a stressor since it activates the same systems involved in responding to an external threat (Hackney, 2006); bouts of exercise increase heart rate, blood pressure and levels of cortisol. Thus, regular activation of stress systems by physical exercise may produce beneficial adaptations such that these systems are able to respond to acute stress more effectively, for example with reduced vigor or shorter duration. This idea has been termed the cross-stressor adaptation hypothesis (Sothmann, 2006).

Previous studies have investigated psychophysiological responses to mentally challenging or psychologically stressful laboratory tasks among healthy individuals with respect to levels of physical fitness. Most of these studies have focused upon cardiovascular reactivity to psychologically stressful tasks with mixed findings. For example, more physically fit individuals have exhibited enhanced reactivity (de Geus et al., 1993), blunted reactivity (Crews and Landers, 1987) or no difference (Steptoe et al., 1990; Blumenthal et al., 1991; Summers et al., 1999; Spalding et al., 2004; Poole et al., 2011) in comparison to their less physically fit counterparts. Further, two recent meta-analyses of studies reached different conclusions regarding cardiovascular reactivity; Jackson and Dishman (2006) reported that physical fitness was associated with a greater cardiovascular response yet quicker recovery, while Forcier et al. (2006) reported that cardiovascular responses were blunted. A limited number of studies have examined cortisol responses to psychological stress. In these studies, physically fit men, older women, and highly active children exhibited blunted cortisol responses to psychological stress in comparison to their less physically active counterparts (Traustadottir et al., 2004; Rimmele et al., 2007; Martikainen et al., 2013). Finally, few studies have reported on emotional responses to stress with respect to levels of physical exercise, and again the findings have been mixed (e.g., Sinyor et al., 1983; Choi and Salmon, 1995; Summers et al., 1999). The discrepancies in findings between studies may be due to methodological differences, including stress induction methods (e.g., speech tasks tend to produce greater psychophysiological responses than mental challenge tasks, Dickerson and Kemeny, 2004) and subject samples studied (e.g., previous studies show evidence of sex differences in responses to psychological stress, Kirschbaum et al., 1999; Kelly et al., 2008; Childs et al., 2010).

In this study we aimed to assess multidimensional responses i.e., physiological (heart rate, blood pressure, salivary cortisol) and psychological (anxiety, positive mood) to a standardized acute psychosocial stressor among young healthy men and women, and to compare stress responses between individuals who reported regular physical exercise and those who did not. To our knowledge no studies have measured multidimensional aspects of stress responses i.e., cardiovascular, hormonal and emotional, in the same individuals which is important to show the existence of relationships between the different modalities. Furthermore, very few studies have examined stress-induced changes in positive and negative mood states with respect to levels of habitual physical exercise. We hypothesized, based on previous reports, that non-exercisers participants would exhibit greater cardiovascular and emotional reactivity, and dampened cortisol responses to stress in comparison to regular exercisers.

## Materials and methods

### Subjects

Participants (*n* = 111) were recruited from the University and surrounding area by flyers and advertisements. They attended the laboratory for an in-person medical screening that included a health and drug use questionnaire and an ECG. Participants were healthy adults, aged 18–32, with body mass index 19–29 kg/m^2^. Exclusion criteria included a current or past year diagnosis of a Major Axis I psychiatric disorder (American Psychiatric Association, 1994), an abnormal electrocardiogram, use of prescription medications including, in women, oral contraceptives (Kirschbaum et al., 1995, 1999), or night shift work. Individuals who smoked >5 cigarettes/week were also excluded as smoking has been shown to alter responses to the TSST (Kirschbaum et al., 1993b; al'Absi et al., 2003; Childs and de Wit, 2009). Information on physical activity was obtained from a questionnaire administered at screening; participants indicated whether they exercised on a regular basis (outside of normal activities including commuting), and how many times per week they exercised. Participants were then classified as sedentary (*n* = 30) or regular exercisers (i.e., ≥1 occasion per week, *n* = 81). Participants were told that the study aim was to examine the effects of verbal tasks on mood and physiology. At the end of the study, they were fully debriefed about the study aims and paid for their participation.

### Procedure

The University of Chicago Hospital's Institutional Review Committee for the use of human subjects approved the study protocols. All participants provided informed consent at a separate orientation session conducted before the study began. Participants completed two sessions at least 48 h apart, one with a stressful task and another with a non-stressful control task, in randomized order. The stressor that we used was the Trier Social Stress Test (TSST; Kirschbaum et al., 1993a). This is a standardized and widely used psychosocial stressor that reliably induces changes in physiological and psychological dimensions (Dickerson and Kemeny, 2004). Participants also completed a non-stressful control task on a separate day to account for diurnal rhythms in mood and physiology (Childs and de Wit, 2009; Het et al., 2009; Childs et al., 2010; Lovallo et al., 2010).

All study procedures were conducted at the Human Behavioral Pharmacology Laboratory at the University of Chicago. Experimental sessions were conducted in testing rooms furnished as a comfortable living area, with an easy chair for relaxing (when participants were not completing study measures), a television and video player, and a desk with a computer for completing study questionnaires. On arrival, participants provided breath and urine samples to detect recent drug or alcohol use (no one tested positive) and then relaxed for 30 min to acclimatize to the laboratory. Baseline measures were obtained and then participants were read instructions for the task to be performed that day. They were allowed 10 min to prepare for each task, at the end of which they were escorted to an adjacent room to perform the task. The TSST consisted of a 5 min speech and 5 min mental arithmetic (serial subtraction) performed before two interviewers who were unknown to the participant and who provided no feedback. There was also a video camera present which projected the participants' image onto a television screen throughout the task. The control task was performed in the absence of a video camera, and involved the participant talking to the research assistant for 5 min about a favorite book, movie or television program, followed by playing a computer game (Solitaire) for 5 min. Before, and at repeated times after the tasks, participants rated their mood, saliva samples were obtained for cortisol analysis, and vital signs were obtained.

### Dependent measures

Saliva samples were collected using Salivette® cotton wads (Sarstedt Inc., Newton, NC) at −30, 10, 20, and 60 min after the tasks and were analyzed by the Core Laboratory at the University of Chicago Hospitals General Clinical Research Center for levels of cortisol (Salimetrics LLC, State College, PA, sensitivity = 0.003 ug/dL). Heart rate (HR) was measured continuously throughout the experimental session (one reading per minute) using a Polar chest band and monitor (Mini-Logger, Mini Mitter/Respironics, Bend, OR). Scores were averaged over consecutive 10 min periods. Blood pressure was measured using a monitor (Critikon Dinamap Plus Vital Signs Monitor, GE Healthcare Technologies, Waukesha, WI) at −30, 0, 10, 20, and 30 min after the tasks. Self-reported mood was measured using the Profile of Mood States questionnaire (POMS, McNair and Droppleman, 1971) at −30 and 0 min after the task.

Personality traits were assessed using the Multidimensional Personality Questionnaire Brief Form (MPQ-BF, Patrick et al., 2002). This questionnaire is an empirically-derived personality instrument with an orthogonal factor structure that yields 11 well-defined primary trait scores and three superfactors termed Positive Emotionality (PEM, extraversion), Negative Emotionality (NEM, neuroticism) and Constraint (CON, behavioral spontaneity).

Some data points were missing due to sample loss or equipment failure, thus there were minor variations in samples sizes between the separate analyses (heart rate *n* = 77; blood pressure *n* = 111; mood *n* = 96; cortisol *n* = 99; personality *n* = 102).

### Statistical analyses

Two markers of stress reactivity were calculated for the outcome measures; (1) peak change from pre-task baseline, which provides a measure of the intensity of the response, and (2) area under the curve relative to the pre-task baseline (AUC, Altman, 1991; Pruessner et al., 2003), which provides information about response duration and recovery of homeostasis after stress exposure. We first confirmed the efficacy of the stress task by comparing responses in the outcome measures between the two tasks using one factor (Task) repeated measures analysis of variance (ANOVA). We also compared responses to the tasks between men and women using two factor (Task*Sex) repeated measures ANOVA, since others have previously reported sex differences in responses to the TSST (Kirschbaum et al., 1999; Kelly et al., 2008; Childs et al., 2010). Sex was included as an additional factor in later analyses for any outcome measures that were significantly influenced by Sex.

We then compared demographic and personality characteristics between the groups using independent samples *t*-test (for continuous variables) and chi-squared analysis (for categorical variables). We also compared baseline measures (average of pre-task scores from each session) between the groups using independent samples *t*-test. We compared responses to the tasks between groups using two-factor repeated measures (Task*Group) ANOVA. All analyses were conducted using SPSS v19 for windows. Finally, we assessed relationships between self-reported frequency of exercise per week and net responses to stress (i.e., response after TSST minus response after control task) using Pearson's correlation coefficient. Effect sizes are reported using partial eta squared (η^2^_ρ_) for analyses of variance; 0.01, 0.06, and 0.14 are considered, respectively, small, medium, and large effect sizes.

## Results

### Demographics

Most participants were of European descent (53%) and in their early twenties (22.1 ± 0.4 years, Table 1). The groups did not differ on any demographic or personality characteristics.

**Table 1:** **Demographic characteristics of study participants**.

	**Non-exercisers**	**Exercisers**
N (male/female)	30 (7/23)	81 (35/46)
Exercise frequency (times/week)	0	3.5 ± 0.2
**RACE (%)**		
European American^*^	30	62
African American	33	17
Other	37	21
Age (years)	21.8 ± 0.7	22.3 ± 0.4
Body mass index (kg/m^2^)	22.0 ± 0.4	22.2 ± 0.2
**CURRENT DRUG USE**		
Caffeine (drinks/week)	8.0 ± 1.8	5.9 ± 0.7
Alcohol (drinks/week)	2.8 ± 0.7	4.0 ± 0.4
Cigarettes (per week)	0.2 ± 0.1	1.1 ± 0.6
**PERSONALITY**		
Positive emotionality	75.0 ± 2.1	74.0 ± 1.7
Negative emotionality	28.2 ± 2.8	26.5 ± 1.6
Constraint	71.2 ± 3.2	70.4 ± 1.9

Values indicate mean ± s.e.m. Asterisks indicate a significant difference between the samples (

* *p < 0.05, Chi-squared analysis)*.

### Baseline measures

Before the tasks began, heart rate was significantly lower among individuals who reported regular exercise [*t*_(80)_ = 2.2; *p* < 0.05, mean difference = 6.3 ± 2.9 bpm] and baseline heart rate was significantly negatively correlated with the frequency of exercise per week (*r* = −0.24; *p* < 0.05) in the whole group. Blood pressure, cortisol and mood did not differ significantly between the groups at baseline (Table 2).

**Table 2:** **Baseline values of physiological measures**.

	**Non-exercisers**	**Exercisers**
Heart rate (bpm)	79.0 ± 2.5	72.4 ± 1.3^*^
Systolic blood pressure (mm Hg)	108.1 ± 2.1	112.1 ± 1.5
Diastolic blood pressure (mm Hg)	65.9 ± 1.6	64.9 ± 0.9
Cortisol (ug/dL)	0.47 ± 0.08	0.38 ± 0.03

Values indicate mean ± s.e.m. Asterisks indicate a significant difference between the samples (

* *p < 0.05, Independent Samples t-test)*.

### Stress reactivity and sex differences

In comparison to the control task, the TSST significantly increased heart rate, blood pressure, and cortisol among all participants (Table 3). The TSST also significantly increased negative affect (Anxiety, Depression, Anger, Confusion) and decreased positive mood states (Friendly, Elation, Positive Mood).

**Table 3:** **Responses to the control task and TSST among all participants**.

	**Control**	**TSST**	***t***
Cortisol (ug/dL)	−0.11 ± 0.03	0.04 ± 0.04	−4.3^***^
Heart rate (bpm)	4.1 ± 0.9	13.0 ± 1.2	7.4^***^
Systolic (mm Hg)	4.1 ± 1.0	12.5 ± 1.1	6.0^***^
Diastolic (mm Hg)	2.3 ± 0.8	8.5 ± 0.7	5.6^***^
Friendliness	−0.06 ± 0.08	−0.55 ± 0.08	4.7^***^
Anxiety	0.01 ± 0.05	0.37 ± 0.07	−4.6^***^
Depression	0.05 ± 0.03	0.14 ± 0.04	−2.2^*^
Anger	0.01 ± 0.04	0.32 ± 0.06	−5.0^***^
Elation	−0.03 ± 0.08	−0.46 ± 0.08	4.3^***^
Confusion	0.05 ± 0.05	0.28 ± 0.06	−3.1^**^
Positive Mood	−0.08 ± 0.09	−0.60 ± 0.09	4.5^***^

Values indicate mean ± s.e.m. Asterisks indicate a significant difference between the samples (

* p < 0.05,

** p < 0.01,

*** *p < 0.001, Independent Samples t-test)*.

Overall, men exhibited greater cortisol responses to the tasks than women [Sex effect: Peak change *F*_(1, 96)_ = 16.7; *p* < 0.001; η^2^_ρ_ = 0.15; AUC *F*_(1, 95)_ = 18.1; *p* < 0.001; η^2^_ρ_ = 0.16] and greater stress-induced increases in systolic blood pressure than women [Task*Sex effect: *F*_(1, 106)_ = 4.2; *p* < 0.05; η^2^_ρ_ = 0.04]. Therefore, Sex was included as a factor in later analyses of these measures. There were no other sex differences in other cardiovascular or emotional responses to the tasks.

### Influence of exercise on stress reactivity

**Figure 1:**
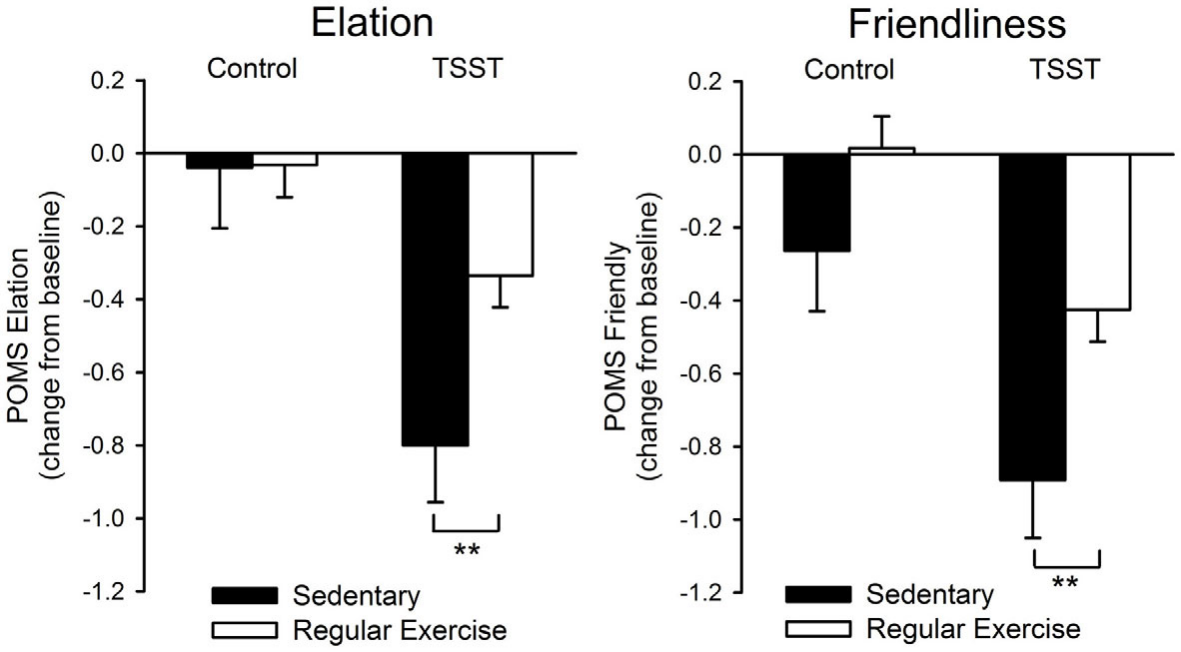
**Change in positive affect (POMS Elation, Friendliness) after the control and TSST tasks among regular exercisers and non-exercisers**. Bars represent mean ± s.e.m. change from pre-task baseline. Asterisks indicate a significant difference between groups (^**^p < 0.01, Independent sample *t*-test).

Heart rate, blood pressure and cortisol reactivity to the stress procedure did not differ between the groups. Individuals who did not regularly exercise exhibited significantly greater decreases in positive affect after stress [Task*Group effect: Elation *F*_(1, 94)_ = 8.38; *p* < 0.01; η^2^_ρ_ = 0.08, Positive Mood *F*_(1, 94)_ = 3.06; *p* = 0.08; η^2^_ρ_ = 0.03, Figure 1]. Overall, regular exercisers also felt more “friendly” after both tasks [Group effect: Friendliness *F*_(1, 94)_ = 4.39; *p* < 0.05; η^2^_ρ_ = 0.05, Figure 1]. Correlation analyses did not show any evidence of significant relationships between the frequency of exercise per week and psychophysiological responses to stress.

## Discussion

This study aimed to compare reactivity to acute stress between healthy individuals who exercise regularly and those who do not. We examined cardiovascular, cortisol and emotional responses to a standardized psychosocial stressor in comparison to a non-stressful control task among healthy male and female participants and in relation to their self-reported levels of regular physical exercise. There were several interesting findings. Overall, heart rate was significantly lower among regular exercisers than sedentary participants, yet cardiovascular reactivity to stress or the tasks overall did not differ between the groups. The groups did not significantly differ in levels of cortisol at baseline or after stress. Also, while subjective mood states did not differ between the groups at baseline, emotional responses to the tasks did differ between the groups; participants who regularly exercised exhibited less of a decline in positive affect after stress than sedentary participants. These findings suggest that regular exercisers may be more resistant to acute stress, which may protect them against future poor health.

The finding that regular exercisers exhibited a smaller decline in positive affect during a stressful situation provides some of the first direct evidence to support that habitual physical activity is associated with stress resilience in healthy individuals. Exercise has been associated with greater well-being in cross-sectional studies of healthy adults but empirical evidence of its beneficial effects is limited. Both groups showed similar levels of positive and negative affect at baseline, and the groups did not differ in personality measures of positivity or negativity. Thus, regular exercise was not associated with higher baseline levels of positive mood, but instead selectively influenced the ability of a stressful situation to diminish positive affect. Interestingly, stress-induced increases in negative affect were similar between the groups. Recent theories have begun to place more importance on the role of positive emotions during stress independent of negative affect (Folkman, 2008). Moreover, positive, but not negative affect, has been linked to a decreased risk of mortality (Moskowitz et al., 2008; Davis, 2009). Thus, an ability to maintain greater positive mood during stress exposure among regular exercisers may serve a protective function, minimizing the accumulation of stress burden with repeated exposures that is linked with the development of disease. Possible explanations for a resistance to stress-induced decreases in positive affect include that individual appraisals of the situation, self-resources, or coping strategies may be more positive among regular exercisers. For example, positive coping strategies have been linked to greater positive affect during stress (Folkman and Moskowitz, 2000, 2004; Lazarus, 2000). Thus, future studies should also look to assess primary and secondary appraisals of stressful situations and the various coping strategies utilized by exercisers and non-exercisers.

Regular exercisers did not exhibit altered reactivity in other components of responses to acute stress which echoes the findings of some previous reports (Blumenthal et al., 1991; Summers et al., 1999; Spalding et al., 2004; Poole et al., 2011). Also, in line with others' findings, heart rate was significantly lower overall among exercisers in comparison to non-exercisers (e.g., de Geus et al., 1990, 1993; Summers et al., 1999), and baseline heart rate was correlated with the frequency of self-reported exercise per week. Thus, although our analysis depended upon self-reported levels of exercise which are more unreliable than objective measures of physical activity, the significant relationship between baseline heart and frequency of self-reported exercise reinforces the validity of our approach.

There were several limitations to the present study. First, as mentioned, we relied upon self-reports of regular physical exercise which can be unreliable. A better method for future studies would be to obtain an objective measure of physical activity, such as that provided by an activity monitor or accelerometer which participants could wear for a week before testing. A second limitation was that the groups were self-selecting, that is there may be a bias introduced when subjects who spontaneously engage in exercise are compared to non-exercising persons. Although there was no difference between regular exercisers and sedentary participants in baseline mood or the personality traits of extraversion and neuroticism, there may be an underlying factor associated with both exercise and stress resilience that is not accounted for in this study. Finally, the proportion of European Americans who reported regular exercise was significantly greater than that reported by other races, and since race has also been shown to influence stress responses (Shen et al., 2004; Chong et al., 2008; Fauvel and Ducher, 2009; Li et al., 2009; Christian et al., 2013), we cannot negate the influence of race upon our findings. Nevertheless, studies of differences in stress responses between races have mainly reported differences in physiological responses to stress, and in this study we did not find any group differences in this measure (Murphy et al., 1992; Saab et al., 1992; Kelsey et al., 2000; Wilcox et al., 2005; Kim, 2008). Thus, in order to conclusively disentangle the effects of race from physical exercise, our findings should be replicated in samples with similar distributions of European Americans.

## Concluding remarks

In conclusion, in this study we assessed multidimensional responses to acute stress in healthy participants who differed in levels of regular exercise. The stress procedure produced a smaller decline in positive mood among the regular exercisers, compared to the sedentary individuals. Responses to the psychosocial stressor used in this study may reflect the way individuals typically respond to daily stressors, suggesting that regular exercisers are more resistant to the emotional effects of acute stress, which in turn, may protect them against diseases related to chronic stress burden.

### Conflict of interest statement

The authors declare that the research was conducted in the absence of any commercial or financial relationships that could be construed as a potential conflict of interest.
